# Health-related quality of life and associated factors in people with HIV: an Irish cohort study

**DOI:** 10.1186/s12955-016-0517-4

**Published:** 2016-08-05

**Authors:** Sherly George, Colm Bergin, Susan Clarke, Grainne Courtney, Mary B. Codd

**Affiliations:** 1 School of Public Health, Physiotherapy and Sports Science, University College Dublin, Woodview House, Belfield, Dublin 4, Ireland; 2 GUIDE Clinic, St James’s Hospital Dublin, Dublin, Ireland; 3 Department of Medicine, Trinity College Dublin, Dublin, Ireland

**Keywords:** HIV, Quality of life, Health-related quality of life, HRQoL, Medical outcomes study HIV Health Survey, MOS-HIV Health survey, Malnutrition universal screening tool, MUST, Medical outcomes study social support survey, MOS SSS

## Abstract

**Background:**

Considering the chronic and debilitating nature of HIV infection, health-related quality of life (HRQoL) is an important patient-reported clinical outcome to better understand the effects of this infection and its treatment on patients’ lives. The purpose of this study was to assess the HRQoL and its association with sociodemographic, behavioural, clinical, nutrition-related factors and social support in an Irish HIV cohort.

**Methods:**

A cross-sectional, prospective study using the Medical Outcomes Study HIV Health survey assessed the 10 dimensions of HRQoL and summarised as Physical Health Summary (PHS) and Mental Health Summary (MHS) scores. Participants were categorised as having good or poor PHS and MHS using the standardised mean score of 50. The variables independently associated with PHS and MHS were identified using multivariable logistic regression models.

**Results:**

Overall, 521 participants completed the HRQoL questionnaire. The median (IQR) PHS and MHS scores were 56 (47–60) and 51 (41–58) respectively. All the covariate groups had lower MHS than PHS. Participants with symptoms of HIV reported the lowest median (IQR) PHS score 44.7 (32.–54.5) and MHS score 36.1 (28.6–48.4). Of the 10 dimensions of HRQoL, the lowest scores were for the energy level and general health. Symptoms of HIV, co-morbidities, social support, employment and ethnicity had independent association with both PHS and MHS. Gender, education, alcohol intake and HIV-complications were associated with PHS. Age, illicit drugs, BMI and malnutrition were associated with MHS. However, CD4 count and viral load were not independently associated with PHS and MHS in multivariable regression models.

**Conclusion:**

Overall, HIV-infected people in this cohort had an average level of HRQoL. However, it is impaired in people with symptoms and co-morbidities, and not independently associated with CD4 and viral load. Alleviating HIV symptoms and preventing co-morbidities are important in managing HIV. Providing psychosocial supports for behaviour modification and return to work or exploring new opportunities will help to improve HRQoL. Healthcare providers and policy makers need to plan and implement programs to routinely assess the HRQoL in a systematic method to facilitate a holistic management of HIV.

## Background

Advances in the management of HIV have resulted in significant improvement in the mortality and morbidity of people living with this infection [1, 2]. As people live longer, their quality of life (QoL) can be adversely affected by the disease progression, issues associated with antiretroviral therapy (ART) and the ageing process [3]. Hence, the ongoing management of HIV aims to improve their physical and psychological well-being and thus health-related quality of life (HRQoL) [1]. In routine clinical practices, assessments of HIV treatment outcomes are usually focussed on the objective clinical parameters including quantification of plasma viral load and CD4 level. However, they reflect the pathological abnormalities related to this disease, but not its effects on QoL [4].

QoL is a multifactorial and multidimensional concept of physical, psychological, and social functioning and well-being, and the impact of a disease and treatment on QoL is referred as HRQoL [5–7]. It is important to assess the HRQoL as a treatment outcome in HIV management as it reflects the patients’ experiences of living with this chronic condition. At the same time, the impact of the factors directly or indirectly related to HIV infection need to be considered due to potential confounding effects of these factors on HRQoL. Also, HRQoL is a subjective concept which is dynamic in terms of person, place and time [8, 9]. Hence, assessing the HRQoL of people living with HIV in different countries is beneficial to compare and better understand the treatment outcomes of this global epidemic condition.

Several previous studies have assessed the HRQoL of people with HIV in different countries. These reports vary in terms of its effects on different dimensions of life and the associated factors [10–19]. The negative effects of HIV on QoL have been reported by many studies [17, 18, 20–23]. On the other hand, one study showed improvement in QoL and they reported this as a result of lifestyle modification after diagnosing HIV [19]. Considering the multifactorial aspect of HRQoL, various studies explored its association with sociodemographic factors, clinical factors, behavioural factors and social support [12, 15, 24–27]. Having symptoms of HIV and co-morbidities, illicit drug use, unemployment and lack of social support were consistently reported to have adverse effects on HRQoL [22, 28–32]. However, reports about the association of age, gender, ethnicity, and ART with HRQoL are inconsistent [3, 12, 22, 24, 26, 27, 29, 33–37]. This may be due to the discrepancies in the research methodologies used in those studies.

Many previous studies have reported separately on the impact of sociodemographic, behavioural and clinical factors and social support on HRQoL, and only few studies addressed the impact of these factors combined. Also, very little is known about the effects of nutritional status on HRQoL, especially when there is a paradigm shift from wasting and underweight to overweight and obesity in HIV-infected people [38, 39]. Overweight and obesity are rapidly growing threat to the health of general population globally and nationally. This increases the risks of developing cardiovascular and endocrine complications and may have adverse effects on their QoL [40]. In a cohort of HIV-infected people on ART, almost half of the participants were reported to be either overweight or obese [41] and increases the risk of developing comorbidities and impairment in HRQoL. Hence constructing statistical models to include all the relevant sociodemographic, behavioural, clinical, nutrition-related factors and social support will facilitate better understanding of significant variables independently associated with HRQoL in this era with advanced management facilities. Also, very little is known about the HRQoL of people with HIV in Ireland. The aim of this study was to assess the HRQoL of people with HIV attending the GUIDE (genito-urinary medicine and infectious diseases) clinic at St James’s hospital, Dublin, and its association with the sociodemographic, behavioural, clinical, nutrition-related factors and social support factors. The GUIDE clinic is one of the 6 HIV specialist centres in Ireland, more than half of outpatient attendees avail care from this clinic, and the study sample characteristics are almost similar to that of the national HIV cohort [42]. The conceptual framework underpinning this study is based on the Wilson and Cleary’s model of assessing health outcomes in terms of biological and physiological variables, symptom status, functional status, general health perception and overall QoL and the causal relationship between these variables [43].

## Methods

### Study design and settings

An observational cross-sectional study was conducted between June 2010 and January 2012 to assess the HRQoL of HIV-infected patients enrolled into the Dublin HIV Cohort study from the GUIDE clinic. The Dublin HIV Cohort study was established in 2006 for 5 years to enrol patients diagnosed with HIV at the three HIV treatment centres in Dublin. A total of 1086 patients were enrolled into the Dublin HIV cohort study from the GUIDE clinic, and 703 participants had one or more follow up visits between June 2010 and January 2012. Of these 703 participants, 555 were approached by one of the researchers and 528 patients consented for the HRQoL assessment with a response rate of 95.1 %. The remaining 148 potential participants were not approached due to the limited number of researchers recruiting participants and the busy clinical settings. Patients with certain physical and mental conditions disabling to complete the questionnaires independently were excluded.

### Data collection methods and measures

Potential participants were identified from the HIV clinic list. They were systematically approached privately on arrival, without disturbing the routines of this very busy clinic. The study was explained to them using the study-specific information leaflet and was given enough time to make an independent decision. Consented participants were interviewed to collect sociodemographic data, behaviour-related data and nutrition-related data using a study-specific case report form (CRF). They then completed three self-administered questionnaires: the Medical Outcomes Study HIV (MOS-HIV) Health Survey; the HIV/AIDS Targeted QoL questionnaire (HAT-QoL) and the Medical Outcomes Study Social Support Survey (MOS SSS) while waiting to be seen by clinician and other healthcare team members. Participants were given assistance to complete questionnaires when needed. Completed questionnaires were briefly verified when returned for any missing data and rechecked with participants to make sure that the missing data were not by chance. This method of data collection helped to reduce missing data and increased the item response rates.

### Outcome variables

The HRQoL was assessed using the MOS-HIV Health Survey which includes 35 closed-ended questions to assess 10 dimensions of HRQoL [44]. Items were scored on Likert scales and total score for each dimension ranged from 0 to 100 with higher the score better the QoL. The Physical Health Summary score (PHS) and Mental Health Summary score (MHS) were derived from the ten dimensions using the algorithm provided in the manual. Using this questionnaire, the minimum and maximum achievable scores are 0 and 100 with a standardised mean score of 50 ± 10 standard deviation for both PHS and MHS [44]. This method of data analysis helps to reduce the dimensionality and improve measurement precision. The MOS-HIV Health Survey is an extensively used and culturally adapted questionnaire and demonstrated good reliability and validity in many previous studies [21, 25, 44–47]. All the items in the general health perception, pain, mental health and energy dimensions are similar to the generic questionnaire SF-36, and hence facilitated comparisons with the available Irish general population QoL scores [48–50].

### Predictor variables

The predictor variables including sociodemographic, behavioural, clinical and nutrition-related variables and perceived social support were collected using the study-specific CRF, HAT-QoL questionnaire [51, 52], Malnutrition Universal Screening Tool (MUST) [53] and MOS SSS questionnaire [54]. These data were based on the relevant information within the 1 year period of the study.

Sociodemographic variables collected using the study-specific CRF were age, gender, ethnicity, education, employment and living condition. Education was categorised as lower education (having no third-level education) and higher education (having a third level education). Based on the past 1 month, employment status (employed or unemployed) was assessed to understand their chances of having some meaningful day-to-day interactions with people. They were considered as employed if working part-time, full-time or involved in any voluntary work. The living condition was categorised as living alone or living with partner and/or with any family members at the time of recruitment. Behavioural variables including sexual activity status, illicit drugs use and use of alcoholic drinks were assessed using the study-specific CRF. Participants were considered as sexually not active if they reported not being sexually active for 1 year or more. Use of alcoholic drink was assessed by asking if the participant had used any alcohol containing drink in the last year and were categorised as yes or no. Illicit drug use was assessed by asking the participants about the use of any illicit drugs in the past year and were categorised as yes or no.

Clinical variables included: the mode of HIV transmission categorised as men having sex with men (MSM) or not MSM, time since HIV diagnosed, HIV-related symptoms perceived by the participants (yes or no), ART status (yes or no), most recent CD4 level (≤350 cells/mm^3^ and >350 cells/mm^3^), most recent viral load (<40 copies/ml and ≥ 40 copies/ml), HIV-associated complications (yes or no) and co-morbidities (yes or no). Any medical condition which could be associated with HIV infection and was diagnosed after having HIV or its treatment was considered as an HIV-associated complication and all the others were considered as co-morbidities. HIV-related symptoms were assessed by asking, “Have you got any symptoms of HIV infection at present?” and were asked to list down if they had any. Participants with one or more symptoms matching with the 20-item HIV symptom index were categorised as having symptoms of HIV [55].

BMI, change in weight over 6 months and risk of malnutrition were the variables included in the nutrition-related factors and were assessed from participants and their clinical records. Risk of malnutrition was assessed using the MUST [53]. Malnutrition risk scores were calculated based on the BMI, % of weight loss over a 6 month period and lack of any nutrition intake for more than 5 days. Based on the total scores obtained, malnutrition risks were assessed as low (total score = 0) or medium/high (total score ≥ 1) risks of malnutrition. Social support-related factors included the perceived social support and disclosure worries. Perceived social support was assessed using the MOS SSS questionnaire. This is a validated questionnaire and used in many previous studies involving people with HIV [21, 54, 56–58]. All the 19 items were scored on a Likert scale of 1 to 5 and an overall social support index was calculated, ranging from 0 to 100, with higher score indicating better availability of social support [54]. Disclosure worries were assessed using the 3 items in the disclosure subscale of the HAT-QoL questionnaire [51, 52]. This questionnaire was also validated and used in many previous studies [26, 59–61]. A total disclosure score ranging from 0 to 100 was calculated. A higher score indicated less disclosure worries.

### Statistical analysis

Data were analysed using the SPSS Software (PASW Statistics18). In univariate analysis *χ*^2^-test for categorical data and Spearman’s correlation for continuous data were performed to assess their association with HRQoL scores. Comparisons of the median PHS and MHS scores of the covariate groups were undertaken using the Mann-Whitney *U* test. One sample *t*-test was used to compare the mean scores with Irish normative values for general health, pain, mental health, and energy dimensions. The PHS and MHS scores were dichotomised as good and poor PHS and MHS groups respectively based on the standardised mean score 50. Since logistic regression analysis identifies the association of predictor variables to a dichotomous outcome variable by controlling the effects of other predictor variables [62, 63] and have been used in many previous studies [28, 31, 32, 47, 64, 65] this statistical test was used to identify potential predictor variables independently associated with PHS and MHS. Because there is no universal consensus in choosing predictor variables in a multivariable logistic regression analysis, we included all the variables with *p* ≤ 0.2 in the univariate analysis and all clinically significant variables regardless of their *p*-values, based on the literature evidence [12, 16, 32]. Significance of the covariates were assessed by the *p*-values (<0.05), odds ratios (OR) and 95 % confidence intervals (CI) for association between predictor variables and good HRQoL. The Hosmer-Lemeshow goodness-of-fit statistic with *p*-value above 0.05 was considered as a well-fitting regression model, and percentage of the variability predicted by model is explained by the Nagelkerke R^2^ [66].

## Results

### Descriptive characteristics of the study participants

A total of 528 people (75.1 % of the accessible population) with HIV, aged 20 to 70 years, participated in this study. Overall, 85.8 % were under 50 years of age and 135 (74.4 %) participants had HIV diagnosed 5 years prior to the study. Participants were mostly males (69 %) and Caucasians (73 %); more than half (54 %) were employed; 38 % of the participants lived alone; and 48 % had third level education. Almost 2/3^rd^ of the participants (65 %) reported them being sexually active at the time of the study. Within the 12 month period prior to the study, a total of 80 (16 %) participants had used illicit drugs and 398 (79 %) used alcoholic drinks. The majority of participants were on ART (89 %), 441 (84 %) had CD4 level above 350/mm^3^ and 391 (74 %) had viral load of < 40 copies/ml. Very few participants (*n* = 86; 16 %) reported of having symptoms of HIV. However, 275 (52.1 %) participants reported to have one or more co-morbid conditions. A total of 281 (53.3 %) participants were overweight or obese, and 60 (11.4 %) had medium or high risk of malnutrition. The majority of participants perceived very good level of social support with a median (IQR) score of 74.2 (49.3–95.3). However, disclosure was a major concern and the median (IQR) score was only 43.8 (25.0–62.5).

### Health-related quality of life

A total of 521 participants completed the MOS-HIV Health Survey questionnaire. The Cronbach’s alpha coefficient calculated to determine the internal consistency reliability of different HRQoL dimensions ranged from 0.83 to 0.91. The HRQoL of these participants in terms of PHS, MHS and 10 dimensional scores are presented in Table 1. The median (IQR) PHS and MHS scores were 56.3 (46.8–60.3) and 50.8 (41.3–57.5) respectively. Of the 10 subscales, the median values of energy/fatigue, general health perception, mental health and quality of life dimensions scores were 75 or below, and were lower than the other dimensions. There was a significantly strong correlation between PHS and MHS (Spearman’s correlation = 0.66; *p* < 0.001). MHS was lower than PHS in all the covariate categories, and majority of these categories had median score lower than the standardised mean score of 50 (Table 2). Since there is no universal consensus on categorisation of continuous data for statistical analysis [67] and the cut-off points used in previous studies were either 25^th^ percentile [28, 32, 47], mean [64] or median [68, 69], the participants in this study were categorised as having good and poor PHS/MHS scores based on the standardised mean score, 50 [44]. Overall, there were 363 (68.8 %) with good PHS and 274 (51.9 %) participants with good MHS.

**Table 1:** HRQoL in patients with HIV: Summary scores and Dimension scores

HRQoL scores	Median (IQR)	Mean (sd)
*Summary Scores*		
Physical Health Summary Score	56.3 (46.8–60.3)	52.1 (11.0)
Mental Health Summary Score	50.8 (41.3–57.5)	48.6 (11.7)
*Dimension scores*		
General Health perception	65.0 (45.0–85.0)	61.4 (26.5)
Physical Functioning	91.7 (66.7–100)	81.8 (25.0)
Role Functioning	100 (50.0–100)	79.3 (37.6)
Social Functioning	100 (80.0–100)	83.6 (27.0)
Cognitive Functioning	85.0 (60.0–95.0)	75.2 (24.9)
Pain	88.9 (55.6–100)	76.7 (25.7)
Mental Health	72.0 (52.0–84.0)	68.0 (22.0)
Energy/Fatigue	60.0 (45.0–80.0)	60.6 (23.3)
Health Distress	80.0 (60.0–95.0)	71.9 (27.4)
Quality of Life	75.0 (50.0–75.0)	68.0 (23.2)

*Abbreviations*: *IQR*: inter quartile range, *sd* standard deviation

**Table 2:** HRQoL in HIV: Covariate categories and comparisons of PHS and MHS median scores

	*n* (%)	PHS: Median (IQR)	*p*	MHS: Median (IQR)	*p*
*Age (years)* ^*a*^	521 (98.7)	−0.065^a^	0.139	0.061^a^	0.165
*Gender*	521 (98.7)	–	0.171	–	0.007
Male	361 (69.3)	56.6 (48.–60.5)	–	51.9 (42.7–57.8)	–
Female	160 (30.7)	55.3 (42.9–60.1)	–	47.2 (36.8–56.4)	–
*Ethnicity*	521 (98.7)	–	<0.001	–	0.025
Caucasian	379 (72.7)	55.5 (44.5–60.1)	–	50.5 (39.3–57.0)	–
Non-Caucasian	142 (27.3)	58.1 (53.0–61.0)	–	51.6 (45.7–58.5)	–
*Education*	517 (97.9)	–	<0.001	–	<0.001
Higher education	247 (47.6)	58.5 (53.1–61.0)	–	53.0 (45.7–58.9)	–
Lower education	272 (52.4)	53.0 (41.5–59.1)	–	48.3 (36.7–56.0)	–
*Employment*	518 (98.1)	–	<0.001	–	<0.001
Employed	282 (54.4)	58.9 (53.7–61.3)	–	54.6 (46.3–60.0)	–
Unemployed	236 (45.6)	50.2 (37.3–58.1)	–	46.1 (35.5–54.2)	–
Living condition	521 (98.7)	–	0.705	–	0.615
Lives with family	323 (62.0)	56.1 (48.1–60.2)	–	53.1 (45.1–58.8)	–
Lives alone	198 (38.0)	56.5 (46.2–60.5)	–	49.2 (39.1–56.4)	–
*Sexual activity status*	495 (93.8)	–	<0.001	–	<0.001
Sexually active	322 (65.1)	57.5 (50.4–60.8)	–	53.0 (44.1–58.6)	–
Sexually not active	173 (34.9)	52.5 (37.8–59.2)	–	47.5 (36.4–54.9)	–
*Current use of illicit drugs*	494 (93.6)	–	0.001	–	<0.001
No	415 (84.0)	56.7 (49.0–60.6)	–	51.9 (42.9–57.6)	–
Yes	79 (16.0)	52.3 (38.3–59.0)	–	44.4 (35.7–55.3)	–
*Use of alcoholic drinks*	494 (93.6)	–	0.017	–	0.007
No	99 (20.0)	53.6 (38.0–59.6)	–	47.7 (34.6–55.5)	–
Yes	395 (80.0	56.8 (48.8–60.4)	–	52.2 (42.3–57.6)	–
*HIV transmission mode*	521 (98.7)	–	0.001	–	0.002
MSM	177 (34.0)	58.1 (50.7–61.0)	–	53.2 (45.3–58.9)	–
Not MSM	344 (66.0)	55.4 (44.7–60.1)	–	49.3 (39.1–56.9)	–
*Time since HIV diagnosed* ^*a*^	521 (98.7)	−0.134^a^	0.002	−0.013^a^	0.774
*HIV symptoms*	521 (98.7)	–	<0.001	–	<0.001
Yes	85 (16.3)	44.7 (32.1–54.5)	–	36.1 (28.6–48.4)	–
No	436 (83.7)	57.5 (50.3–60.8)	–	52.6 (44.8–58.5)	–
*Current ART status*	521 (98.7)	–	0.096	–	0.168
Yes	436 (88.9)	56.1 (46.6–60.3)	–	50.6 (40.9–57.5)	–
No	58 (11.1)	57.6 (52.6–60.7)	–	53.8 (44.3–58.1)	–
*Recent CD4 level*	521 (98.7)	–	0.003	–	<0.001
≤ 350cells/mm^3^	87 (16.7)	51.7 (37.6–59.6)	–	46.3 (36.0–53.9)	–
> 350cells/mm^3^	434 (83.3)	56.7 (48.9–60.4)	–	51.8 (42.3–57.9)	–
*Recent viral load*	521 (98.7)	–	0.112	–	0.008
< 40 copies/ml	413 (79.3)	56.5 (48.8–60.4)	–	51.2 (42.6–57.9)	–
≥ 40 copies/ml	108 (20.7)	55.2 (42.9–60.1)	–	47.9 (35.0–55.8)	–
*HIV complications*	521 (98.7)	–	<0.001	–	0.006
No	441 (84.6)	57.0 (49.2–60.7)	–	51.4 (42.4–57.7)	–
Yes	80 (15.4)	49.2 (36.6–56.6)	–	47.5 (35.0–55.1)	–
*Co-morbidities*	521 (98.7)	–	<0.001	–	<0.001
Yes	271 (52.0)	51.7 (38.5–58.8)	–	47.1 (35.7–55.0)	–
No	250 (48.0)	58.9 (54.2–61.1)	–	54.5 (46.3–59.7)	–
*BMI (kg/m* ^*2*^ *)* ^*a*^	520 (98.5)	−0.003^a^	0.948	0.022^a^	0.622
*Risk of malnutrition*	517 (97.3)	–	0.001	–	0.004
Low risk	457 (88.4)	56.6 (49.1–60.4)	–	51.5 (42.4–57.6)	–
High risk	60 (11.6)	47.8 (35.9–58.8)	–	45.3 (33.7–55.5)	–
*Overall Social Support* ^*a*^	509 (96.4)	0.299^a^	<0.001	0.386^a^	<0.001
*Disclosure issues* ^*a*^	490 (92.8)	0.062^a^	0.182	0.026^a^	0.561

*Abbreviations*: *PHS* physical health summary score, *MHS* mental health summary score, *IQR* inter quartile range

^a^Spearman’s correlation

Using the one-sample *t*-test, the total scores for the comparable dimensions of this study were compared with the existing Irish normative values for general population [50]. HIV participants’ scores were significantly lower for the general health perception (61.4 vs 73.8, *p* < 0. 001, 95 % CI: −14.7 to −10.2), mental health (67.9 vs 64.8, *p* < 0.001, 95 % CI: −11.7 to −7.9) and energy (60.6 vs 64.8, *p* < 0.001, 95 % CI: −6.3 to −2.2). There was no significant difference in the pain scores.

The median PHS and MHS scores of the covariate categories were compared and presented in Table 2. The PHS and MHS scores were significantly higher in participants who: had higher education (*p* < 0.001), were employed (*p* < 0.001), were sexually active (*p* < 0.001), not currently using illicit drugs (*p* = 0.001), did not have symptoms of HIV (*p* < 0.001), had CD4 level > 350cells/mm^3^ (*p* < 0.005), did not have HIV complications (*p* < 0.01), did not have co-morbidities (*p* < 0.001), and had low risk of malnutrition (*p* < 0.005). Significantly higher MHS score was reported by male participants (*p* = 0.007) and participants with a recent viral load < 40 copies/ml (*p* = 0.008). Perceived social support was moderately correlated with PHS and MHS scores (*p <* 0.001) and time since HIV diagnosed had a weak correlation with PHS score (*p* = 0.002), but not with MHS (Table 2). However, there was no correlation between disclosure worries and HRQoL score.

The association of the predictor variables with the PHS and MHS categories were explored using univariate analysis and multivariable logistic regression analyses (enter method) and are presented in Tables 3 and 4. The variables significantly associated with both PHS and MHS scores in the multivariable models were symptoms of HIV, comorbidities, employment, ethnicity and social support (Table 3). Male gender, higher education, not using alcoholic drinks and not having HIV-associated complications were associated with good PHS, whereas age, no current use of illicit drugs, BMI and low risk of malnutrition were associated with MHS. Many variables significantly associated with PHS and MHS in the univariate analyses were not significant in the multivariable models (Table 4). The Hosmer-Lemeshow goodness-of-fit test *p*-values were 0.402 and 0.349 for the PHS and MHS models constructed respectively suggestive of well-fitting models. It is assumed that 47 % of the variability in the PHS (Nagelkerke R^2^ = 0.47) and 38 % of the variability in the MHS (Nagelkerke R^2^ = 0.38) is explained by these models.

**Table 3:** HRQoL in HIV patients: Multivariable Logistic Regression Models for PHS and MHS

	Good PHS		Good MHS	
Variables	OR (95 % CI)	*p*	OR (95 % CI)	*P*
Age (Years)	1.00 (0.97–1.03)	0.919	1.05 (1.02–1.08)	0.001
Gender (Male)	2.47 (1.22–4.98)	0.012	1.76 (0.96–3.24)	0.068
Ethnicity (Non-Caucasian)	3.55 (1.63–7.73)	0.001	2.11 (1.12–3.99)	0.021
Education (Higher education)	2.33 (1.32–4.12)	0.004	1.10 (0.68–1.80)	0.697
Employment (Employed)	4.02 (2.27–7.14)	<0.001	2.12 (1.28–3.50)	0.003
Living condition (Lives with family)	0.81 (0.45–1.47)	0.491	0.70 (0.42–1.16)	0.166
Sexually active (Yes)	1.22 (0.70–2.11)	0.478	1.04 (0.63–1.71)	0.881
Current use of illicit drugs (No)	1.55 (0.76–3.16)	0.224	2.53 (1.31–4.89)	0.006
Current use of alcoholic drinks (No)	2.00 (1.01–3.98)	0.047	0.69 (0.39–1.25)	0.223
MSM (Yes)	0.95 (0.47–1.92)	0.890	0.69 (0.38–1.26)	0.227
Time since HIV diagnosed (Years)	1.01 (0.97–1.06)	0.579	1.03 (0.98–1.07)	0.258
Symptoms of HIV (Yes)	3.49 (1.77–6.89)	<0.001	3.56 (1.79–7.10)	<0.001
On ART (Yes)	3.11 (0.96–10.11)	0.059	2.47 (0.95–6.42)	0.063
CD4 count (>350 cells/mm^3^)	1.22 (0.63–2.39)	0.558	1.54 (0.83–2.86)	0.170
Viral load (<40 copies/ml)	1.65 (0.72–3.79)	0.240	1.73 (0.81–3.72)	0.158
HIV-associated complications (No)	1.97 (1.01–3.82)	0.046	1.13 (0.60–2.12)	0.708
Co-morbid conditions (No)	2.29 (1.29–4.07)	0.004	2.46 (1.50–4.02)	<0.001
BMI (Kg/m^2^)	0.97 (0.90–1.03)	0.283	0.92 (0.86–0.97)	0.004
Malnutrition risk (Low)	1.73 (0.75–3.97)	0.198	2.25 (1.02–4.97)	0.045
Social support (Total score 0–100)	1.03 (1.02–1.04)	<0.001	1.03 (1.02–1.04)	<0.001
Disclosure worries (Total score 0–100)	1.00 (0.99–1.01)	0.608	1.01 (1.00–1.02)	0.105

Good PHS: PHS score > 50; Good MHS: MHS score >50

*Abbreviations*: *OR* odds ratio, *CI* confidence interval

**Table 4:** Univariate analysis and Multivariable logistic regression analysis models for PHS and MHS

	Good PHS (Total PHS score > 50)				Good MHS (Total MHS score > 50)			
	Univariate analysis		Multivariable analysis		Univariate analysis		Multivariable analysis	
Variables	OR (95 % CI)	*p*	OR (95 % CI)	*p*	OR (95 % CI)	*p*	OR (95 % CI)	*p*
*Age (years)*	0.984 (0.96–1.01)	0.13	1.00 (0.97–1.03)	0.919	1.024 (1.00–1.05)	0.017	1.05 (1.02–1.08)	0.001
*Gender*								
*Male*	1.546 (1.04–2.30)	0.031	2.47 (1.22–4.98)	0.012	1.611 (1.11–2.34)	0.013	1.76 (0.96–3.24)	0.068
Female (Ref)	–	–	–	–	–	–	–	–
*Ethnicity*								
Non-Caucasian	2.53 (1.57–4.09)	<0.001	3.55 (1.63–7.73)	0.001	1.23 (0.84–1.81)	0.295	2.11 (1.12–3.99)	0.021
Caucasian (Ref)	–	–	–	–	–	–	–	–
*Education*								
Higher education	3.581 (2.37–5.40)	<0.001	2.33 (1.32–4.12)	0.004	1.75 (1.23–2.48)	0.002	1.10 (0.68–1.80)	0.697
Lower education (Ref)	–	–	–	–	–	–	–	–
*Employment*								
Employed	5.524 (3.65–8.43)	**<0.001**	4.02 (2.27–7.14)	**<0.001**	3.327 (2.32–4.78)	**<0.001**	2.12 (1.28–3.50)	**0.003**
Unemployed (Ref)	–	–	–	–	–	–	–	–
*Living condition*								
Lives with family	1.037 (0.71–1.52)	0.851	0.81 (0.45–1.47)	0.491	1.038 (0.73–1.48)	0.838	0.70 (0.42–1.16)	0.166
Lives alone (Ref)	–	–	–	–	–	–	–	–
*Sexual activity status*								
Sexually active	2.626 (1.77–3.91)	<0.001	1.22 (0.70–2.11)	0.478	1.768 (1.22–2.57)	0.003	1.04 (0.63–1.71)	0.881
Sexually not active (Ref)	–	–	–	–	–	–	–	–
*Current use of illicit drugs*								
No	2.237 (1.37–3.66)	0.001	1.55 (0.76–3.16)	0.224	2.229 (1.36–3.66)	0.002	2.53 (1.31–4.89)	0.006
Yes (Ref)	–	–	–	–	–	–	–	–
Use of alcoholic drinks								
No	0.742 (0.47–1.18)	0.209	2.00 (1.01–3.98)	0.047	0.496 (1.36–3.66)	0.002	0.69 (0.39–1.25)	0.223
Yes (Ref)	–	–	–	–	–	–	–	–
*HIV transmission mode*								
MSM	1.96 (1.29–2.99)	0.002	0.95 (0.47–1.92)	0.89	1.678 (1.16–2.43)	0.006	0.69 (0.38–1.26)	0.227
Not MSM (Ref)	–	–	–	–	–	–	–	–
*Time since HIV diagnosed*	0.956 (0.93–0.99)	0.004	1.01 (0.97–1.06)	0.579	1 (0.97–1.03)	0.987	1.03 (0.98–1.07)	0.258
*HIV symptoms*								
No	5.221 (3.20–8.53)	**<0.001**	3.49 (1.77–6.89)	**<0.001**	5.294 (3.042–9.21)	**<0.001**	3.56 (1.79–7.10)	**<0.001**
Yes (Ref)	–	–	–	–	–	–	–	–
*Current ART status*								
No	1.578 (.83–3.02)	0.167	3.11 (0.96–10.11)	0.059	1.316 (0.76–2.29)	0.33	2.47 (0.95–6.42)	0.063
Yes (Ref)	–	–	–	–	–	–	–	–
*Recent CD4 level*								
> 350cells/mm^3^	2.029 (1.26–3.26)	0.003	1.22 (0.63–2.39)	0.558	2.166 (1.35–3.48)	0.001	1.54 (0.83–2.86)	0.17
≤ 350cells/mm^3^ (Ref)	–	–	–	–	–	–	–	–
*Recent viral load*								
< 40 copies/ml	1.471 (0.94–2.30)	0.089	1.65 (0.72–3.79)	0.24	1.511 (0.99–2.31)	0.058	1.73 (0.81–3.72)	0.158
≥ 40 copies/ml (Ref)	–	–	–	–	–	–	–	–
*HIV complications*								
No	3.097 (1.90–5.04)	<0.001	1.97 (1.01–3.82)	0.046	1.615 (1.00–2.61)	0.051	1.13 (0.60–2.12)	0.708
Yes (Ref)	–	–	–	–	–	–	–	–
*Co-morbidities*								
No	3.873 (2.57–5.85)	**<0.001**	2.29 (1.29–4.07)	**0.004**	2.529 (1.78–3.60)	**<0.001**	2.46 (1.50–4.02)	**<0.001**
Yes (Ref)	–	–	–	–	–	–	–	–
*BMI (kg/m* ^*2*^ *)*	1.014 (0.97–1.06)	0.533	0.97 (0.90–1.03)	0.283	0.982 (0.95–1.02)	0.369	0.92 (0.86–0.97)	0.004
*Risk of malnutrition*								
Low risk	3.069 (1.78–5.31)	<0.001	1.73 (0.75–3.97)	0.198	2.48 (1.41–4.38)	0.002	2.25 (1.02–4.97)	0.045
High risk (Ref)	–	–	–	–	–	–	–	–
*Overall Social Support*	1.022 (.02–1.03)	**<0.001**	1.03 (1.02–1.04)	**<0.001**	1.025 (1.02–1.03)	**<0.001**	1.03 (1.02–1.04)	**<0.001**
*Disclosure issues*	0.996 (0.99–1.00)	0.321	1.00 (0.99–1.01)	0.608	1.002 (1.00–1.01)	0.577	1.01 (1.00–1.02)	0.105

*Abbreviations*: *OR* odds ratio, *CI* confidence interval, *PHS* physical health summary score, *MHS* mental health summary score

The bolded *p*-values correspond to the variables that are significant in both the univariate and multivariable analyses

## Discussion

Overall, the HRQoL of the patients with HIV in this cohort were within the average level. This was a group of HIV-infected people availing of advanced HIV management, with good immune function, viral suppression and majority were under 50 years of age. Hence, their functioning, well-being and overall HRQoL could be expected to be almost similar to the general population. However, this cohort had lower scores on their general health perception, energy and mental health, compared to the general population in Ireland [50] indicating that HIV-infected peoples’ physical and mental health functioning and well-being are still impaired, though there is significant improvement in mortality and morbidity [1, 2]. On the other hand, participants in this study reported better HRQoL compared to a small previous Irish study in late 1990s using the MOS-HIV Health survey [11]. Currently, people are diagnosed in early stages of HIV, treated with ART before their immune functions deteriorate and thus are spared many HIV-associated complications. As a result, the impact of this illness on their physical health and functioning is substantially lower than a decade ago. In that study, the participants reported lowest score of 38 for the health distress dimension, indicating that they were discouraged and frightened by HIV. A notable reduction in health distress in our study may be related to the transformation of this once lethal condition to a treatable illness, and lessened the uncertainty about life and anxiety about future.

Having symptoms of HIV, complications and co-morbidities were the most significant clinical variables independently associated with HRQoL. The relapsing and remitting nature of HIV illness could affect functionality and life satisfaction leading to impaired QoL. Symptoms of HIV have been reported as being associated with HRQoL in many previous studies also [16, 27, 28, 30, 31, 70, 71]. The most common symptoms reported by the participants in our study were sleep disturbances, lack of energy, abdominal discomfort, lipodystrophy and depression. All these may be due to the unfavourable effects of HIV-associated stigma, discrimination, disclosure and other psychosocial issues. Disclosure was substantially a major concern for majority of the participants irrespective of the duration of being diagnosed with HIV. Providing training programs such as Yoga, Meditation, Mindfulness and Counselling to reduce emotional stress and to improve mental health, psychological functioning and well-being may help them to manage these issues [72–75]. According to Creswell et al. mindfulness meditation trainings improved HIV-infected patients’ adherence to medication and buffered the immune function [75].

As evident in the literature, participants with good social support had better HRQoL compared to their counterparts [21, 47, 58, 76, 77]. Like any other chronic illness, these patients also need someone to care for their physical, psychological and social needs. However, the fear of discrimination and rejection by family members, relatives and friends prevent them from disclosing their HIV status resulting in mental conflicts and isolation. This may reduce the number of people they can rely on to share and discuss their anxieties and concerns. Some people are even reluctant to seek employment because of the worry of disclosing to their HIV status to employer and co-workers.

The association of BMI and malnutrition with MHS as a risk factor suggests that increase in BMI may lead to impairment in HRQoL. When explored further, the median MHS was lower in the underweight (BMI < 18.5 kg/m^2^) and obese (BMI ≥ 30 kg/m^2^) groups compared to the normal weight (BMI 18.5 to < 25 kg/m^2^) and overweight (BMI ≥ 25 kg/m^2^ to < 30 kg/m^2^) groups. Obesity may be due to the lack of physical activities and poor lifestyle behaviours. This may lead to mental health issues including depression as reported by Harrington et al. in the overweight and obese group based on the SLÁN study in general Irish population [78]. They reported that obese participants were less likely to engage in healthy lifestyle behaviours and had higher chances of depression. Another reason could be the anxiety and worries related to the body disfigurations and the abnormal fat distribution as reported by Verolet et al. in a recent study [79].

The association of employment and HRQoL were already reported in many previous studies [16, 22, 31, 65, 80]. In general, unemployment has an adverse effect on psychological well-being and QoL of an individual due to the feeling of worthlessness, loneliness, financial limitations and poor standard of living. On the other hand, people with HIV may be reluctant to go back to wok or seek new job because of the stress and misconceptions related to disclosure of their HIV status to their employers and colleagues. However, it was not clear whether the unemployment in our study was due to HIV infection as no further data related to their unemployment were collected. Future studies to explore the reasons for unemployment in HIV-infected people are warranted to plan programmes to support them to get jobs, engage in voluntary activities and other interventions to improve their HRQoL.

Our findings show that the non-Caucasians have better HRQoL compared to their Caucasian counterparts. HRQoL is a subjective construct and is influenced by different factors of human life directly or indirectly related to health. In our study more than 90 % of the non-Caucasians were from SSA countries and the majority of them migrated to Ireland during the Celtic tiger period. Better healthcare services compared to their home countries [81] and the well-established social welfare system in Ireland may have helped them to have a high level HRQoL.

Age was a protector of good MHS, and was not associated with PHS. Participants in this study were mostly under 50 years of age and the chances of impairments in their physical functioning due to ageing are less. As people with chronic illnesses live longer, they learn to cope and adjust with the illness, especially when their physical functioning is less affected. Since majority of the older participants had HIV infection for more than 10 years, their ability to cope and adjust with this illness will be better than their younger counterparts. Another factor is that the older participants have more life experiences and thus higher tolerance of the stresses of life by acquiring effective stress management skills, especially when living with a chronic illness like HIV.

The most important limitation is the cross-sectional nature of this study. Hence the exact direction of the associations between predictor variables and HRQoL cannot be established. A follow-up study in near future will be of beneficial to explore and better understand the associations. Recruitment of participants from the outpatient clinics and thereby excluding hospitalised patients and those who cannot or were not attending outpatient clinics is another limitation of this study. This may have even contributed to the high ceiling effects on the role functioning and social functioning dimensions of HRQoL. Nevertheless, statistically controlling the confounding effects of more than twenty factors related to HIV-infected people’s lives and the percentages of HRQoL variabilities explained by the two statistical models are strengths of this study. To our knowledge, in Ireland, this is the first study with large sample size and complexity to assess the HRQoL of people with HIV.

## Conclusions

There is still impairment in the HRQoL of people with HIV in this era with advanced management facilities. The routinely assessed CD4 count and viral load do not reflect the effects of HIV on their HRQoL. Hence, regular assessment of HRQoL using reliable methods will help to better understand the effects of HIV and its treatment on people’s lives and to facilitate a holistic management of this chronic illness. Alleviating symptoms of HIV and preventing comorbid conditions and obesity are important in managing HIV. Providing psychosocial support to return to work or exploring new opportunities will help to improve HRQoL.

## Abbreviations

ART, antiretroviral therapy; BMI, body mass index; CI, confidence interval; CRF, case report form; HAT-QoL, HIV/AIDS targeted QoL; HIV, human immunodeficiency virus; HRQoL, health-related quality of life; IQR, inter quartile range; IVD, intravenous drug users; MHS, mental health summary score; MOS-HIV, medical outcomes study HIV; MOS SSS, medical outcomes study social support survey; MSM, men having sex with men; MUST, malnutrition universal screening tool; OR, odds ratio; PHS, physical health summary score; QoL, quality of life; SF-36, short form 36
